# Organic Fluorescent Dyes Supported on Activated Boron Nitride: A Promising Blue Light Excited Phosphors for High-Performance White Light-Emitting Diodes

**DOI:** 10.1038/srep08492

**Published:** 2015-02-16

**Authors:** Jie Li, Jing Lin, Yang Huang, Xuewen Xu, Zhenya Liu, Yanming Xue, Xiaoxia Ding, Han Luo, Peng Jin, Jun Zhang, Jin Zou, Chengchun Tang

**Affiliations:** 1 School of Materials Science and Engineering, Hebei University of Technology, Tianjin, 300130, P.R. China; 2 Hebei Key Laboratory of Boron Nitride Micro and Nano Materials, Tianjin 300130, P.R. China; 3 Department of Physics, Central China Normal University, Wuhan, 430079, P. R. China; 4 Materials Engineering, The University of Queensland, St Lucia, QLD 4072, Australia; 5 Centre for Microscopy and Microanalysis, The University of Queensland, St Lucia, QLD 4072, Australia

## Abstract

We report an effective and rare-earth free light conversion material synthesized via a facile fabrication route, in which organic fluorescent dyes, i.e. Rhodamine B (RhB) and fluorescein isothiocyanate (FITC) are embedded into activated boron nitride (αBN) to form a composite phosphor. The composite phosphor shows highly efficient Förster resonance energy transfer and greatly improved thermal stability, and can emit at broad visible wavelengths of 500–650 nm under the 466 nm blue-light excitation. By packaging of the composite phosphors and a blue light-emitting diode (LED) chip with transparent epoxy resin, white LED with excellent thermal conductivity, current stability and optical performance can be realized, i.e. a thermal conductivity of 0.36 W/mk, a Commission Internationale de 1'Eclairage color coordinates of (0.32, 0.34), and a luminous efficiency of 21.6 lm·W^−1^. Our research opens the door toward to the practical long-life organic fluorescent dyes-based white LEDs.

Great attentions have been paid on the solid-state lighting during the past decade since the development of InGaN-based light-emitting diodes (LEDs) because of their many excellent properties including environmental friendliness, long life-time, energy saving, and fast response times^1^,^2^,^3^,^4^. Accordingly, their many applications have been proposed and demonstrated^5^,^6^. Traditionally, the high-quality white LEDs could be obtained by mixing the short-wavelength blue light from a blue LED chip, which does not cause damages to package materials and illuminated bodies, with the excited re-emission long-wavelength light from a phosphor mixture^7^,^8^,^9^,^10^. In general, to obtain wavelength conversion layer with blue-light excitable, high quantum efficiency, and small thermal quenching, the rare earth contained phosphors are widely used. However, the use of rare earth ions has led to serious environmental concerns and high cost^11^. Therefore, the search for a method of rare-earth free, low-cost, high thermal stability, and scalable fabrication of high-quality wavelength conversion layer is highly important.

Porous materials made of inorganic materials, especially porous boron nitride, are a promising class of hosts for applications in air and water purifications, catalysis, optics, and gas separation^12^,^13^,^14^,^15^,^16^,^17^, due to their high specific surface area, pore volume, thermal conductivity, and chemical durability^18^,^19^,^20^,^21^. Recently, some porous materials have been used as host media for molecules and complexes to form composite fluorescence exhibiting excellent optical functionalities^22^,^23^,^24^. In the organic fluorescent dye impregnated composites, the high surface area and adjustable mesoscopic structures of the porous hosts offer opportunities for controlling the local environments of occluded dye molecules. Effective Förster resonance energy transfer (FRET) between the dyes could be observed in the case of nanocomposites using micelles, zeolites and nanofibers as hosts due to the restricted dimension and the spectral overlap between the donor and the acceptor^25^,^26^. Therefore, loading fluorescent dyes on solid hosts is an effective way to accelerate the FRET process between donor and acceptor organic dyes. Especially, FRET has been applied in wavelength conversion layers for light emitting diodes (LEDs)^27^. Energy-transfer procedures add additional benefits for improving the emitting performance of LEDs, and for producing beam modulators with a high Stokes shift, and so forth^28^,^29^,^30^.

In this study, activated boron nitride (αBN) was employed as an ideal host for fluorescent dye molecule due to its high thermal conductivity, optically transparency, numerous structural defects, hydroxyl, and organic surface groups^31^. The structural defects, hydroxyl and organic surface groups on the αBN surfaces make these materials interact well with the guest molecules, preventing the elution of the molecules from αBN^12^,^31^,^32^. Here, rhodamine B (RhB) and fluorescein isothiocyanate (FITC) were chosen as the fluorescent dye since they have interesting excitation and emission wavelengths, and are highly luminescent and photostable^33^,^34^. Accordingly, the high quality greenish-orange-emitting (RhB & FITC)/αBN (αBN containing RhB and FITC) composites were synthesized. Utilization of αBN with high thermal conductivity as the host for those dyes could greatly enhance their thermal and optical stability as well as their fluorescent efficiency. Consequently, the epoxy resin mixed with the (FITC & RhB)/αBN, showing improved thermal conductivity of 0.36 W/mk and tensile stress of 174 MPa, were coated onto a blue LED to fabricate the (FITC & RhB)/αBN-based whit LED. The resultant white LED exhibited excellent current stability, Commission Internationale de 1'Eclairage (CIE) color coordinates of (0.32, 0.34), and luminous efficiency of 21.6 lm·W^−1^, suggesting its promising application for an illumination light source.

## Results

### Fabrication route of the white LED

**Figure 1:**
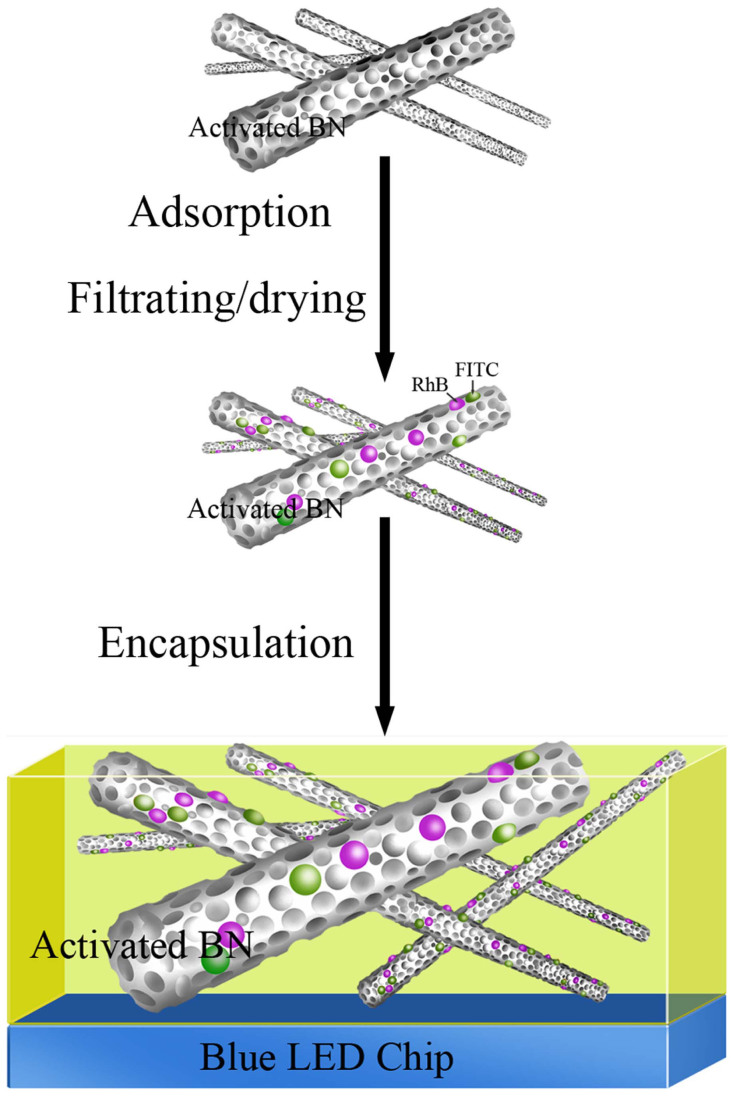
Rare-earth free, low-cost, green, and scalable fabrication route to the high-quality (FITC & RhB)/αBN-based white LEDs.

A novel green and rare-earth free method was developed to synthesize high-quality white LEDs, as illustrated in Figure 1. Firstly, the as-synthesized αBN fibers were introduced into the aqueous solution of FITC and RhB to obtain the (FITC & RhB)/αBN composites (αBN containing RhB and FITC) via an adsorption process (Step 1). After a filtering and drying process, the concentration of the dyes in the filtrate was estimated from absorption measurements after adsorption of the dye molecules onto αBN. The results showed almost 100% of the two dyes were bound to the αBN matrix (Figure S1). Secondly, the composite was mixed with transparent epoxy resin and then the resultant product was encapsulated on a blue LED chip to produce the high-quality (FITC & RhB)/αBN-based white LED (Step 2). To better understand the emitting performance of the (FITC & RhB)/αBN-based white LEDs, αBN and (FITC & RhB)/αBN (αBN-supported FITC and RhB) were fabricated and investigated for comparison, as shown in Figure S2.

### Photoluminescence test of (FITC & RhB)/αBN

**Figure 2:**
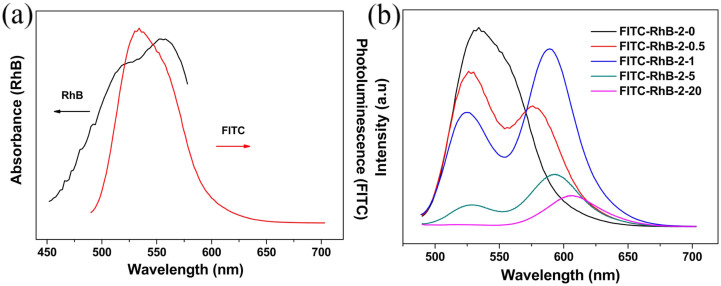
(a) Absorption spectrum of RhB/αBN (100 mL, 4 mg/L) and emission spectrum of FITC/αBN (100 mL, 2 mg/L), respectively. (b) Fluorescence spectra of FITC (100 mL, 2 mg/L) in the presence of different concentration of RhB (100 mL) in the solid media of αBN, respectively.

With the aim of obtaining white light with high color rendering index, the orange-yellow-emitting RhB and green-emitting FITC were chosen as the conversion materials. As shown in Figure 2a, the emission spectrum of the intermediate product, i.e. FITC/αBN (donor) in the range of 500–550 nm matches well the absorption spectrum of RhB/αBN (acceptor). The spectral over-lap between the emission band of FITC/αBN and absorption band of RhB/αBN indicates that efficient FRET process will take place in the (FITC & RhB)/αBN composites^22^,^35^.

**Figure 3:**
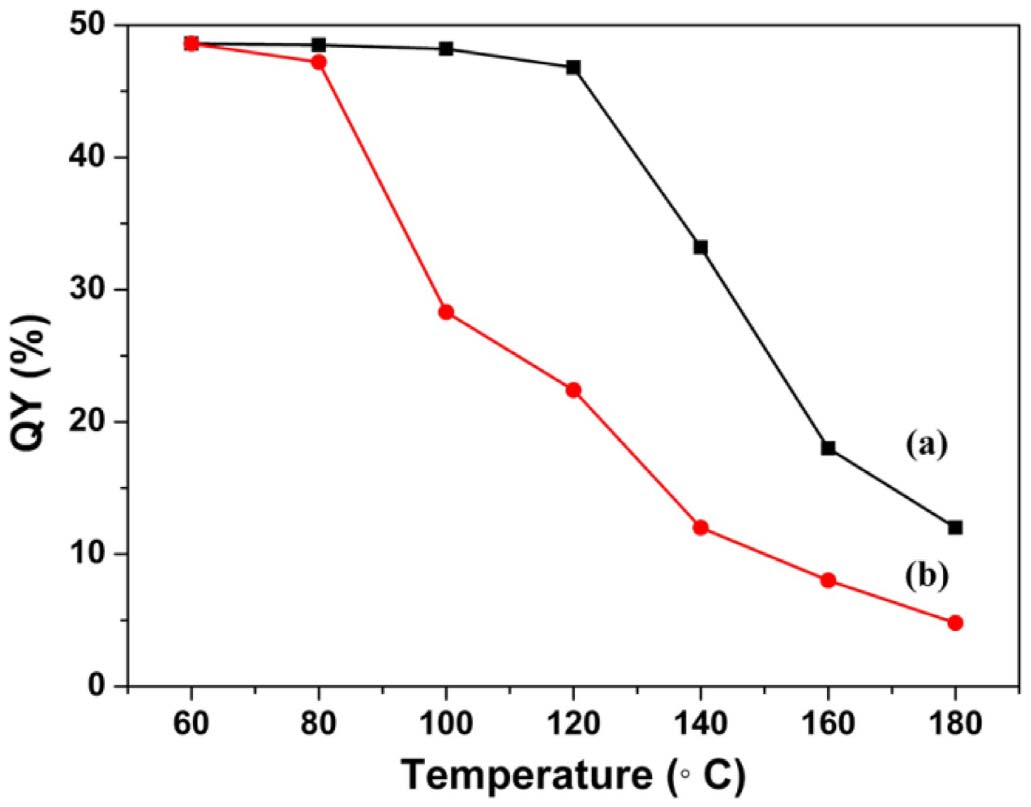
PL quantum yields of (a) (FITC & RhB)/αBN, (b) FITC & RhB as function of the temperature.

Figure 2b shows the fluorescence spectra of (FITC & RhB)/αBN prepared by αBN adsorbing the two dyes at varying concentration ratios in aqueous solution. We chose 466 nm as the excitation wavelength because this wavelength matches well with the blue LED chip as well as the adsorption of FITC at 466 nm is strong. The concentration of FITC is fixed at 2 mg/L, and the concentration of RhB varies from 0 to 20 mg/L. Here, we use FITC-RhB-C-D to represent the mixed (FITC & RhB)/αBN system (αBN adsorbing the given concentration of TIFC and RhB in the aqueous solution). C and D denote the fixed concentration of FITC (2 mg/L) and the various concentrations of RhB from 0 to 20 mg/L, respectively. For example, FITC-RhB-2-5 represents a (FITC & RhB)/αBN composite obtained by 100 mg of αBN adsorbing 2 mg/L (100 mL) FITC and 5 mg/L (100 mL) RhB. The spectra of the (FITC & RhB)/αBN composites contain two broad emissions in the range of 500–550 nm and 570–650 nm, respectively. The broad green band corresponds to the fluorescence emission of FITC, while the orange-yellow is related to the emission of RhB. It is found that the fluorescence emission of FITC was gradually quenched with the increasing concentration of RhB. The fluorescence emission of RhB became intense and reached the maximum intensity value when the concentration of RhB increased to 1 mg/L. Accordingly, the absorption efficiency and Photoluminescence (PL) quantum yields (QY) of the as-prepared (FITC & RhB)/αBN (FITC-RhB-2-1) are high up to 79.2% and 48.6% under the excitation wavelength of 466 nm, respectively. It is noteworthy that the quantum yields of the (FITC & RhB)/αBN composite was still retained after annealing at 140°C for 24 h in air, while the sample of (FITC & RhB) without BN was only 22.4% after annealing at the same condition, as shown in Figure 3. In addition, the composites were also irradiated for 7 days by the short-wavelength blue light (466 nm) from a blue LED chip under a forward bias current of 20 mA. We found that after irradiation, the composite exhibits almost no change in the quantum yields, as depicted in Figure S3. These results suggest that the as-prepared (FITC, RhB)/αBN has improved thermal stability and photo-stability.

### Optical properties of the white LED

**Figure 4:**
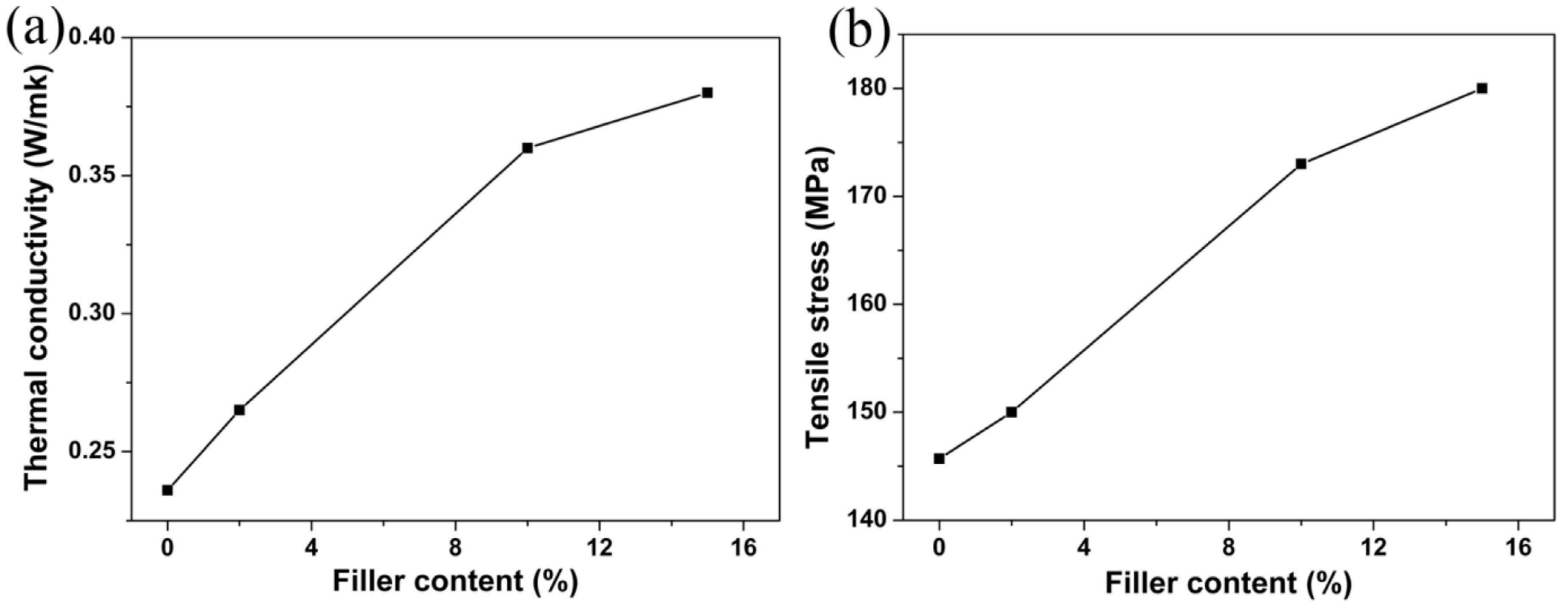
(a) Thermal conductivity (b) tensile stress of the (RhB & FITC)/αBN-based white LED as function of the (FITC & RhB)/αBN: epoxy resin weight ratio of 0.2:10 to 1.5:10.

**Figure 5:**
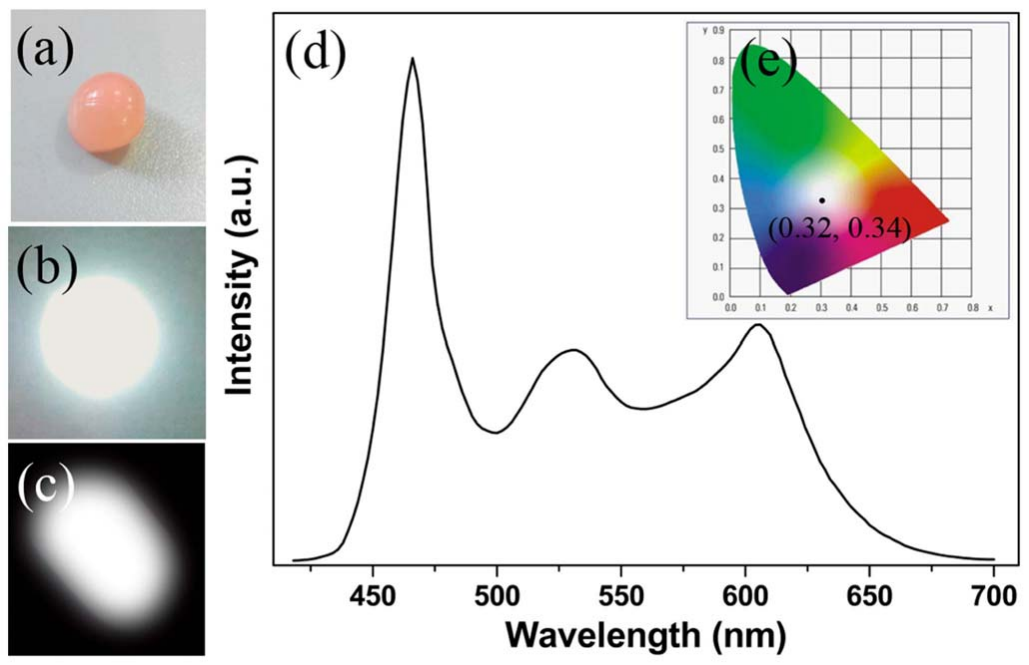
Photographs of (a) (FITC & RhB)/αBN-based white LEDs, (b) and (c) the corresponding white light-emitting LEDs operated at 60 mA and 20 mA, respectively. (d) Spectrum of the white-emitting (FITC & RhB)/αBN-based LED under a blue LED with 466 nm wavelength. (e) The corresponding CIE color coordinates of white LED.

To fabricate the white LED with high performances, the transparent epoxy resin within the appropriate amount of (FITC & RhB)/αBN were encapsulated together on a blue LED chip (a given mass ratio of (RhB & FITC)/αBN: epoxy resin = 1:10). The numerous structural defects, hydroxyl and organic surface groups make the (FITC & RhB)/αBN composites display hydrophilicity which is similar with the epoxy resin. This feature is helpful for the (FITC & RhB)/αBN homogeneously dispersing in the epoxy resin. It is worth noting that the αBN can enhance the mechanical and thermal properties of the polymer during the LED encapsulation, while preserving their electrical insulation and optical transparency^36^. Compared to the pure epoxy resin, the thermal conductivity and tensile stress of the polymer containing (FITC & RhB)/αBN are high up to 0.36 W/mk and 174 MPa, respectively, as depicted in Figure 4(a,b). There results indicate that the heat produced by the blue LED chip can be efficiently transmitted to reduce the working temperature of the white LED even under high forward bias currents. As shown in Figure 5a, the color of the assembled LED device is pinky due to the combination of the orange-yellow FITC and the rose RhB. Obviously, the dispersion of the (FITC & RhB)/αBN in the epoxy resin is homogeneous. Figure 5b and Figure 5c show images of the (FITC & RhB)/αBN-based white LED under forward bias currents of 60 and 20 mA, respectively. When the white LED was operated at 60 mA, the emission was very strong and it was difficult to obtain a sharp image of the fabricated white LED, as evident in Figure 5b. In order to obtain a clear emission image, it was operated at 20 mA (commercial white LEDs are operated at 20 mA), and the white LED still generated bright white light. Figure 5d shows the emission spectra of the white LED under forward bias current of 20 mA. Three emission bands were located at 466, ~533, and ~605 nm, respectively, which displayed a broad visible spectrum that appears white light to the eye.

The assembled white LED exhibits the CIE color coordinates of (0.32, 0.34) (Figure 5e), a color temperature (T_c_) of 6078 K, and a color rendering index (R_a_) of 91.9 under a forward bias current of 20 mA. In comparison with commercial YAG:Ce^3+^-based white LED showing a related low color rendering index (R_a_ < 85) as well as CdSe QD- and Sr_3_SiO_5_:Ce^3+^, Li^+^-based white LED (R_a_ < 90.1)^10^,^37^,^38^, our (RhB & FITC)/αBN-based white LED shows excellent values which are both related to white color. Luminous efficiency of the fabricated white LED was 21.6 lm·W^−1^.

**Figure 6:**
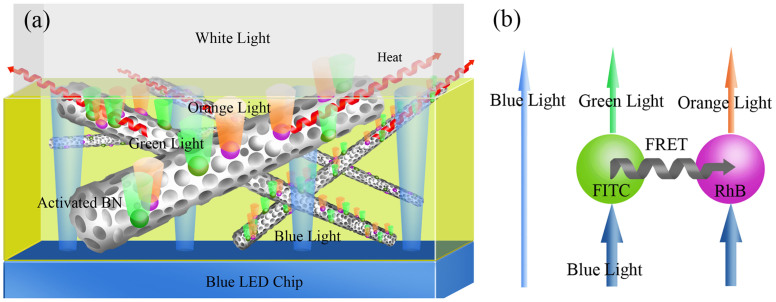
Working principle behind white LEDs (inset showing schematic diagram of the wavelength conversion layer).

Figure 6 illustrates the basic principle behind the white (RhB & FITC)/αBN-based LED. The white LED was obtained by coating a blue LED chip with a wavelength conversion layer, which consisted of the epoxy resin mixed with the (RhB & FITC)/αBN. The (RhB & FITC)/αBN composites were excited by the blue light effectively and emitted intense long-wavelength light, i.e. green and orange light. Additionally, the efficient energy transfer between organic fluorescent dyes could further enhance the emitted intensity of orange light and improve the optical performance of the composites (in inset of Figure 6), which agrees with the observation of Figure 2b. The green and orange light combined with the transmitted blue light to obtain mixed white light. The output intensity ratio of blue light and converted long-wavelength light determines the overall chromatic performance of a white LED^39^. Furthermore, some blue light was absorbed by epoxy resin or (RhB & FITC)/αBN and then transferred to heat. The as-transferred heat and the heat produced by the blue LED chip can be efficiently transmitted due to high thermal conductivity of αBN, which is favourable to increasing the service life of white LEDs.

**Figure 7:**
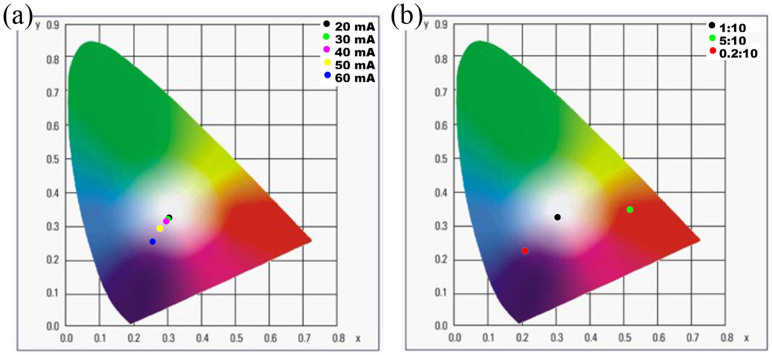
CIE diagrams of the (RhB & FITC)/αBN-based white LED of (a) under various forward currents (direct current 20, 30, 40, 50, and 60 mA), (b) as function of the (FITC & RhB)/αBN: epoxy resin weight ratio of 0.2:10 to 5:10.

With the aim of understanding the optical stability of the assembled (RhB & FITC)/αBN-based white LED, the change in optical property of the LED device under various applied forward currents was investigated by using CIE color coordinate and T_c_, as show in Figure 7a. Obviously, with increasing the forward current, the CIE color coordinate changed slightly in the white color range, indicating the optical stability of the white LED device. Besides, it also exhibited a good color temperature stability upon an increase in forward bias current (T_c_ = 6078 K at 20 mA → 14600 K at 60 mA). The CIE color coordinate changed slightly at the operated temperature of 140°C for 24 h, which agrees well with the QY observation in Figure 3. Moreover, the luminous efficiency and color rendering index of the fabricated white LED were almost remained (21.5 lm·W^−1^ and 91.9) when it was operated at 20 mA for 7 day, respectively. The optical behaviour in the present study may be ascribed to the beneficial effect of αBN medium and the high thermal conductivity of BN/resin, which may enhance the stability of fluorescent lights of the dyes.

The optical properties of the (RhB & FITC)/αBN-based white LEDs were also evaluated by using different mass ratios of (RhB & FITC)/αBN to the epoxy resin, as shown in Figure 7b. When the initial mass ratio of (RhB & FITC)/αBN to the epoxy resin increased from 0.2:10 to 5:10, the white LED exhibited very large variation in the CIE color coordinates and the T_c_, respectively. For a given ratio of (RhB & FITC)/αBN: epoxy resin (1:10), it exhibited the most excellent optical properties (CIE color coordinate: (0.32, 0.34)), T_c_: 6078 K) compared with other two LEDs samples (T_c_ = 1913 K at 5:10, or → 132255 K at 0.2:10). This means that various types of white LEDs with adjustable correlated T_c_ can be simply generated from (FITC & RhB)/αBN-based LEDs by optimizing the mass ratio of (RhB & FITC)/αBN to epoxy resin.

## Discussion

Based on the above-mentioned experimental observations, it is rationalized that αBN is an ideal host for improving the energy transfer efficiency between donor and acceptor organic dyes and thermal and photo stability. The surface hydroxyl organic groups and structural defects of αBN can well interact with the guest dye molecules by the electrostatic force and/or hydrogen bond since the RhB and FITC have hydroxyl groups as well as RhB are positively charged^31^,^32^. This interaction can restrict the vibration of dye molecules, thus reducing the energy loss, and consequently making these molecules be effectively dispersed in the pore channel and/or on the surface of the αBN fibers as well as enhancing their thermal stability and photo-stability^22^,^26^,^40^,^41^,^42^,^43^. According to Wang *et al.*^22^, the energy transfer between organic fluorescent dyes in porous hosts was more efficient than that in liquid solution. Therefore, when FITC and RhB are introduced to the pore channel and/or surface of αBN, the αBN host can control the local environments of the dyes effectively and more effective energy transformation process will be taken place between the two dyes. In addition, the αBN host does not weaken the emission flux of either dyes due to its highly optical transparency (h-BN has a wide band gap of ~5.8 eV).

In addition, the numerous structural defects and hydroxyl and organic surface groups make the (FITC & RhB)/αBN composites display similar hydrophilicity with the epoxy resin, which is helpful for the (FITC & RhB)/αBN homogeneously dispersing in the epoxy resin(Figure 4a). This result contributes to the uniformity of the light that is emitted from the LED device (Fig. 4c), depending on the viewing angle^40^.

It is worth noting that after packaging of the (FITC & RhB)/αBN composites with expoxy resin, the emission spectrum of RhB had a significant red-shift, from 594 nm (Fig. 3) to 605 nm (Fig. 4). According to Song et al.^44^, the phosphor exhibited longer wavelength emissions in epoxy resin than that in liquid solution because the surrounding medium affected the excitation binding energy of the phosphor to some extent. Agglomeration of (FITC & RhB)/αBN within the epoxy resin may also give rise to an additional red-shift of the emission band. The different degree of agglomeration in the white LEDs can explain why different concentration of (FITC & RhB)/αBN in the epoxy resin can vary the color temperature of the white LEDs (Fig. 6b). Moreover, the wavelength conversion layer has no interference with either of the blue light and converted long-wavelength light due to the αBN and the epoxy resin being optically transparency as well as protecting the embedded dye molecules from environmental perturbations. Especially, the heat transferred via light conversion layer adsorbing the blue light and the heat produced by the blue LED chip can efficiently transmit to the atmosphere via αBN to reduce the temperature of the blue LED and conversion layer due to high thermal conductivity of αBN^22^,^23^, which is important for increasing the optical stability and service life of the whit LED.

In summary, the eminent greenish-orange-emitting (RhB & FITC)/αBN have been fabricated via a rare-earth free, low-cost, and facile fabrication route. When using αBN as a host, effective FRET was observed due to the restricted dimension and the spectral overlap between the donor and the acceptor dye. The synthesized composites showed improved thermal stability compared to the pure Organic Fluorescent Dyes. When the epoxy resin mixed with (RhB & FITC)/αBN was coated onto a blue LED chip, the white LED with a high optical property was obtained. The (RhB & FITC)/αBN-based white LED exhibited a CIE color coordinates of (0.32, 0.34), a luminous efficiency of 21.6 lm·W^−1^, and current stability (up to 60 mA). The αBN with high thermal conductivity could effectively improve the thermal stability of the white LED to increase its service life. Additionally, the white LED showed a broad range of white lights with tunable color temperature as well as acceptable for general lighting, indicating that the combination of (RhB & FITC)/αBN and epoxy resin in the LEDs can be a good wavelength conversion layer to obtain white light sources with excellent optical properties.

## Methods

### Synthesis of the (FITC and RhB)/αBN

Activated BN (αBN) as host material are obtained by adopting the two-step reaction of boric acid and melamine with poly(ethylene glycol)-poly(propylene glycol)-poly(ethylene glycol), which was thoroughly discussed in our previous work^25^. The αBN (100 mg) were heated at 200°C for 6 h to remove the water molecules adsorbed on surface of αBN and then immediately transferred to a flask and allowed to cool to room temperature under N_2_ atmosphere. An aqueous solution (100 mL) of FITC (2 mg/L) and RhB (0, 0.5, 1, 5, 20 mg/L) was added into the flask with stirring for 3 h, respectively. After adsorption, the as-obtained samples ((RhB & FITC)/αBN) were filtered and washed thoroughly with the deionized water to ensure the unadsorbed organic fluorescent dye molecules were removed completely.

### Fabrication of the (FITC and RhB)/αBN-based white LEDs

Firstly, the as-prepared (FITC and RhB)/αBN composites were mixed with transparent epoxy resin together and then encapsulated on a blue LED chip (the emission wavelength of 466 nm, non-epoxy molding packages, Seoul Optodevice Co., Ltd., Korea) to form a white LED device. Fabrication details are provided in the Supporting Information.

### Characterization

The structures of the samples were examined and analyzed using X-ray powder diffraction (XRD, BRUKER D8 FOCUS). Fourier transform infrared (FTIR) spectra were recorded on a Nicolet 7100 spectrophotometer between 400 and 4000 cm^−1^. Field emission scanning electron microscopy (SEM, HITACHI S-4800) was employed to characterize the morphology of the products. The specific surface area measurement by using N_2_ adsorption/condensation was carried out at 77 K on an AutoSorb iQ-C TCD analyzer (5-point Brunauer-Emmett-Teller (BET) analysis). Thermal conductivity was measured by means of a thermal measurement apparatus (LW-9091IR-Series). Tensile stress was recorded on a mechanical analyzer (3119-506, INSTRON). A double beam UV/vis spectrophotometer (HITACHI, U-3900H) was used to determine the concentration of dye samples. The excitation and emission spectra were measured by a Hitachi F-7000 spectrophotometer at room temperature. An integrating sphere was used in the assessment of luminous efficiencies.

## Author Contributions

L.J. and T.C. conceived and designed the experiments. L.J., H.Y., X.X., L.Z., X.Y., D.X., Z.J. and J.P. performed the experiments and analyzed the data. Z.J. and L.H. preformed the SEM characterization. L.J., T.C. and L.J. wrote the manuscript. All authors discussed and commented on the manuscript.

## Supplementary Material

Supplementary Information Supporting Information

## References

[b1] Xie R. J., Hirosaki N., Mitomo M., Sakuma K. & Kiumra N. Wavelength-tunable and thermal stable Li-α-sialon: Eu^2+^ oxynitride phosphors for white light-emitting diodes. Appl. Phys. Lett. 89, 241103 (2006).

[b2] Kimura N. *et al.* Extrahigh color rendering white light-emitting diode lamps using oxynitride and nitride phosphors excited by blue light-emitting diode. Appl. Phys. Lett. 90, 051109 (2007).

[b3] Park J. K., Choi K. J., Kim K. N. & Kim C. H. Investigation of strontium silicate yellow phosphors for white light emitting diodes from a combinatorial chemistry. Appl. Phys. Lett. 87, 031108 (2005).

[b4] Xie R. J., Hirosaki N., Li Y. Q. & Takeda T. Rare-earth activated nitride phosphors: synthesis, luminescence and applications. Materials 3, 3777 (2010).

[b5] Wang S., Wang K., Chen F. & Liu S. Design of primary optics for LED chip array in road lighting application. Opt. Express 19, A716–A724 (2011). 21747539 10.1364/OE.19.00A716

[b6] Lo Y. C., Chen C. C., Chou H. Y., Yang K. Y. & Sun C. C. Design of a bike headlamp based on a power white-light-emitting diode. Opt. Eng. 50, 080503 (2011).

[b7] Schlotter P., Schmidt R. & Schneider J. Luminescence conversion of blue light emitting diodes. Appl. Phys. A 64, 417-418 (1997).

[b8] Sakuma K. *et al.* Warm-white light-emitting diodes with yellowish orange SiAlON ceramic phosphor. Opt. Lett. 29, 2001–2003 (2004). 15455760 10.1364/ol.29.002001

[b9] Xie R. J., Hirosaki N., Kimura N., Sakuma K. & Mitomo M. 2-phosphor-converted white light-emitting diodes using oxynitride/nitride phosphors. Appl. Phys. Lett. 90, 191101 (2007).

[b10] Xie R. J., Hirosaki N., Mitomo M., Takahashi K. & Sakuma K. Highly efficient white-light emitting diodes fabricated with short-wavelength yellow oxynitride phosphors. Appl. Phys. Lett. 88, 101104 (2006).

[b11] Zhang H. X. & Rui Y. K. Determination of trace elements, heavy metals and rare earth elements in corn seeds from Beijing by ICP-MS Simultaneously. E-J Chem. 8, 782–786 (2011).

[b12] Li J. *et al.* Porous boron nitride with high surface area: hydrogen storage and water treatment. Nanotechnology 24, 155603–155609 (2013). 23518673 10.1088/0957-4484/24/15/155603

[b13] Lei W. W., Portehault D., Liu D., Qin S. & Chen Y. Porous boron nitride nanosheets for effective water cleaning. Nat. Commun. 4, 1777 (2013). 23653189 10.1038/ncomms2818

[b14] Tang C. C., Bando Y., Ding X. X., Qi S. R. & Golberg D. Catalyzed collapse and enhance hydrogen storage of BN nanotubes. J. Am. Chem. Soc. 124, 14550–14551 (2002). 12465961 10.1021/ja028051e

[b15] Wood G. L. & Paine R. T. Aerosol synthesis of hollow spherical morphology boron nitride particles. Chem. Mater. 18, 4716–4718 (2006).

[b16] Weng Q. H., Wang X. B., Zhi C. Y., Bando Y. & Golberg D. Boron nitride porous microbelts for hydrogen storage. ACS Nano 7, 1558–1565 (2013). 23301807 10.1021/nn305320v

[b17] Weng Q. H., Wang X. B., Bando Y. & Golberg D. One-step template-free synthesis of highly porous boron nitride microsponges for hydrogen storage. Adv. Energy Mater. 4, 1301525–1301533 (2014).

[b18] Paine R. T. & Narula C. K. Synthetic routes to boron nitride. Chem. Rev. 90, 73 (1990).

[b19] Sichel E. K., Miller R. E., Abrahams M. S. & Buiocchi C. J. Heat capacity and thermal conductivity of hexagonal pyrolytic boron nitride. Phys. Rev. B. 13, 4607–4611 (1976).

[b20] Tang C. C. *et al.* Thermal conductivity of nanostructured boron nitride materials. J. Phys. Chem. B 110, 10354–10357 (2006). 16722739 10.1021/jp0607014

[b21] Zhi C. Y., Bando Y., Tang C. C., Kuwahara H. & Golberg D. Large-scale fabrication of boron nitride nanosheets and their utilization in polymeric composites with improved thermal and mechanical properties. Adv. Mater. 21, 2889–2893 (2009).

[b22] Wang L. Z., Liu Y. L., Chen F., Zhang J. L. & Anpo M. Manipulating energy transfer processer between Rhodamine 6G and Rhodamine B in different mesoporous hosts. J. Phys. Chem. C 111, 5541–5548 (2007).

[b23] Wirnsberger G., Dang Y. P., Scott B. J., Chmelka B. F. & Stucky G. D. Mesostructured materials for optical applications: from low-k dielectrics to sensors and lasers. Spectrochim. Acta A 57, 2049–2060 (2001). 10.1016/s1386-1425(01)00503-0 11666084

[b24] Chang Z. X. & Kevan L. Photoionization of tetraphenylporphyrin in mesoporous SiMCM-48, AlMCM-48, and TiMCM-48 molecular sieves. Langmuir 18, 911–916 (2002).

[b25] Kumar C. V. & Chaudhari A. Probing the donor and acceptor dye assemblies at the galleries of α-zirconium phosphate. Microporous Mesoporous Mater. 41, 307–318 (2000).

[b26] Lee K. J., Oh J. H., Kim Y. & Jang J. Fabrication of Photoluminescent-Dye Embedded Poly(methyl methacrylate) Nanofibers and Their Fluorescence Resonance Energy Transfer Properties. Adv. Mater. 18, 2216–2219 (2006).

[b27] Kim T. H. *et al.* Enhanced electrophosphorescence via highly efficient energy transfer from conjugated polymer. Appl. Phys. Lett. 86, 171108 (2005).

[b28] Adronov A. *et al.* Light harvesting and energy transfer in laster-dye-labeled poly(aryl ether) dendrimers. J. Am. Chem. Soc. 122, 1175–1185 (2000).

[b29] Reineke S. *et al.* White organic light-emitting diodes with fluorescent tube efficiency. Nature 459, 234–239 (2009). 19444212 10.1038/nature08003

[b30] Baldo M. A. *et al.* Forrest Highly efficient phosphorescent emission from organic electroluminescent devices. Nature 395, 151–154 (1998).

[b31] Li J. *et al.* Activated boron nitride as an effective adsorbent for metal ions and organic pollutants. Sci. Rep. 3, 3208 (2013). 24220570 10.1038/srep03208 PMC3826095

[b32] Li J., Jin P. & Tang C. C. Cr(III) adsorption by fluorinated activated boron nitride: a combined experimental and theoretical investigation. RSC Adv. 4, 14815–14821 (2014).

[b33] Obata M. *et al.* Synthesis and photophysical properties of rhodamine B dye-bearing poly(isobutyl methacrylate-co-2,2,2-trifluoroethyl methacrylate) as a temperature-sensing polymer film. J. Polym. Sci., Part A: Polym. Chem. 45, 2876–2885 (2007).

[b34] Zareba-Grodz I., Pazik R., Hermanowicz K., Strek W. & Maruszewski K. Preparation and optical properties of hybrid coatings based on epoxy-modified silane and rhodamine B. J. Lumin. 119-120, 148–152 (2006).

[b35] Berggren M., Dodabalapur A., Slusher R. E. & Bao Z. Light amplification in organic thin films using cascade energy transfer. Nature 389, 466–469 (1997).

[b36] Zhi C. Y., Bando Y., Tang C. C., Huang Q. & Golberg D. Boron nitride nanotubes: functionalization and composites. J. Mater. Chem. 18, 3900–3908 (2008).

[b37] Jang H. S. *et al.* White Light-Emitting Diodes with Excellent Color Rendering Based on Organically Capped CdSe Quantum Dots and Sr_3_SiO_5_:Ce^3+^, Li^+^ Phosphors. Adv. Mater. 20, 2696–2702 (2008). 25213892 10.1002/adma.200702846

[b38] Masenelli B. *et al.* YAG:Ce nanoparticle lightsources. Nanotechnology 24, 165703 (2013). 23535555 10.1088/0957-4484/24/16/165703

[b39] Tran N. T. & Shi F. G. Studies of phosphor concentration and thickness for phosphor-based white light-emitting-diodes. J. Lightwave Technol. 26, 3556–3559 (2008).

[b40] Gutiérrez M. C., Hortigüela M. J., Ferrer M. L. & Monte F. Highly fluorescent Rhodamine B nanoparticles entrapped in hybrid glasses. Langmuir 23, 2175 (2007). 17279710 10.1021/la0621057

[b41] Fujii K., Iyi N., Sasai R. & Hayashi S. Preparation of a novel luminous heterogeneous system: rhodamine/coumarin/phyllosilicate hybrid and blue shift in fluorescence emission. Chem. Mater. 20, 2994–3002 (2008).

[b42] Sasai R., Itoh T., Ohmori W., Itoh H. & Kusunoki M. Preparation of characterization of rhodamine 6G/alkyltrimethylammonium/laponite hybrid solid materials with higher emission quantum yield. J. Phys. Chem. C 113, 415–421 (2009).

[b43] Lewkowicz A. *et al.* Concentration-dependent fluorescence properties of rhodamine 6G in titanium dioxide and silicon dioxide nanolayers. J. Phys. Chem. C 116, 12304–12311 (2012).

[b44] Song H. & Lee S. Red light emitting solid state hybrid quantum dot-near-UV GaN LED devices. Nanotechnology 18, 255202 (2007).

