# Supplementary material for: Organic Fluorescent Dyes Supported on Activated Boron Nitride: A Promising Blue Light Excited Phosphors for High-Performance White Light-Emitting Diodes
Source: Sci Rep. 2015 Feb 16;5:8492. doi: 10.1038/srep08492 (PMC4329563; doi:10.1038/srep08492)
Supplement: Supplementary Information — Supporting Information [file srep08492-s1.doc]

**Supplementary Information**

**Organic Fluorescent Dyes Supported on Activated Boron Nitride: A Promising Blue Light Excited Phosphors for High-Performance White Light-Emitting Diodes**

Jie Li^1,2^, Jing Lin^1,2,4^,*, Yang Huang^1,2^, Xuewen Xu^1,2^, Zhenya Liu^1,2^, Yanming Xue^1,2^, Xiaoxia Ding^3^, Han Luo^1,2^, Peng Jin^1,2^, Jun Zhang^1,2^, Jin Zou^4,5^ and Chengchun Tang^1,2^,*

^1^ School of Materials Science and Engineering, Hebei University of Technology, Tianjin, 300130, P.R. China, ^2^ Hebei Key Laboratory of Boron Nitride Micro and Nano Materials, Tianjin 300130, P.R. China, ^3^ Department of Physics, Central China Normal University, Wuhan, 430079, P. R. China, ^4^Materials Engineering, The University of Queensland, St Lucia, QLD 4072, Australia, ^5^ Centre for Microscopy and Microanalysis, The University of Queensland, St Lucia, QLD 4072, Australia

*Phone: +86-22-60202660; fax: +86-22-60202660; e-mail: tangcc@hebut.edu.cn (C.C. Tang); linjing@hebut.edu.cn (J. Lin)

*Phone: +86-22-60202660; fax: +86-22-60202660; e-mail: tangcc@hebut.edu.cn (C.C. Tang)


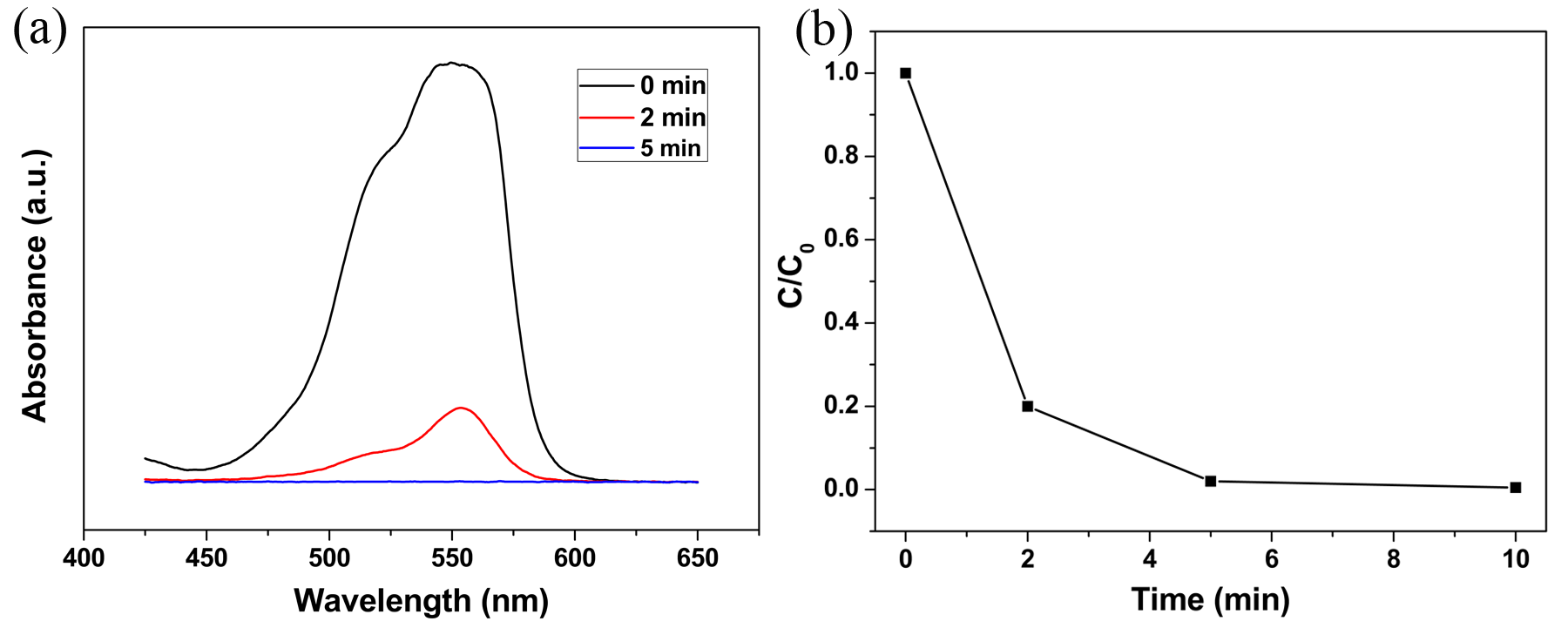


**Figure S1** (a) UV-Vis absorption spectra of the aqueous RhB solution (20 mg/L, 100 ml) at different time intervals after adding 100 mg αBN. (b) Adsorption rate of the mixed FITC (2 mg/L) and RhB (20 mg/L) aqueous solution (100ml, αBN: 100 mg).

The concentration of the dyes in the filtrate was estimated from absorption measurements after adsorption of the dye molecules onto αBN. The results showed almost 100% of the two dyes were bound to the αBN matrix, as shown in Figure S1.


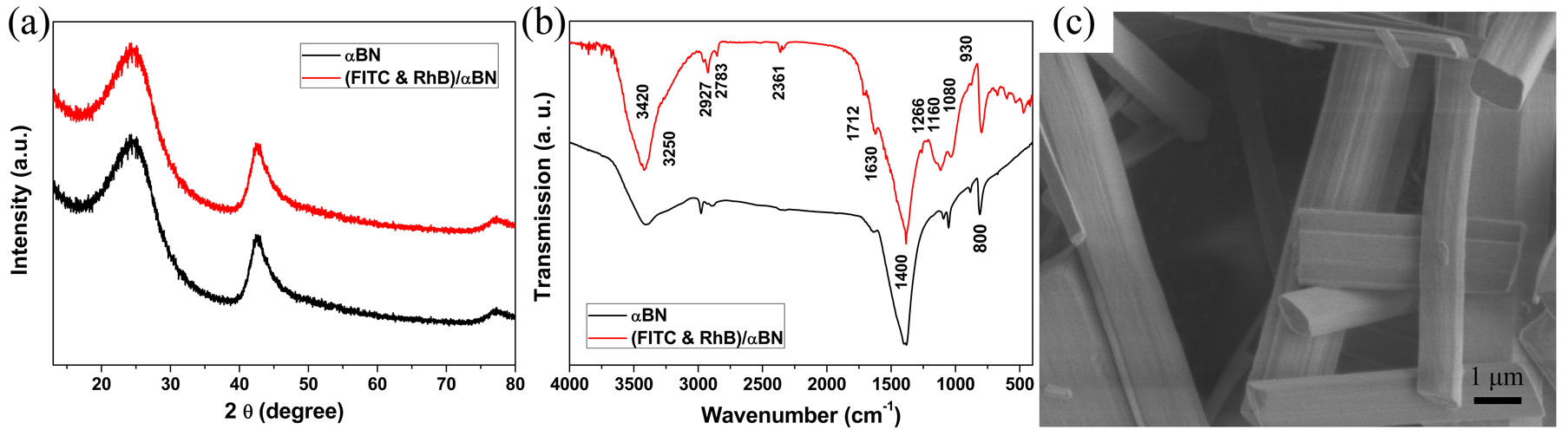


**Figure S2** (a) XRD patterns and (b) FTIR spectra for αBN and (FITC & RhB)/αBN, (c) SEM image of (FITC & RhB)/αBN, respectively.

XRD patterns of both the as-synthesized αBN and (FITC & RhB)/αBN indicate broad diffraction peaks at 2*θ* of 20-30° and 40-45°, which can be indexed to the (002) and (100) planes of the hexagonal boron nitride (h-BN), as displayed in Figure S2a. No other different peaks were observed, indicating that there was no crystalline damage on the samples during the adsorption process. Their structures are close to that of the turbostratic BN materials. The amine groups and hydroxyl groups on the two samples can be observed by the FTIR spectra (3250 cm^-1^ and 3420 cm^-1^), as depicted in Figure S2b. Compared with the FTIR spectrum of αBN, that of (FITC & RhB)/αBN possessed additionally remarkable surface bonds, such as benzene aromatic rings (1712 cm^-1^) and -COOH (1260 cm^-1^). Moreover, all peaks in the FTIR spectra of (FITC & RhB)/αBN became stronger except for the two characteristic peaks of B-N bond stretching (~1400 cm^-1^), B-N-B angle bending (~800 cm^-1^). These results suggest that the FITC and RhB were adsorbed onto the αBN. As shown in Figure S2c, the (FITC & RhB)/αBN exhibited a ribbon-like microstructure with length of ~80 μm, thickness of ~500 nm and width of ~1.0 μm. In addition, the specific surface area of (FITC & RhB)/αBN from the multi-point BET analysis reduced to 10 m^2^/g.

**
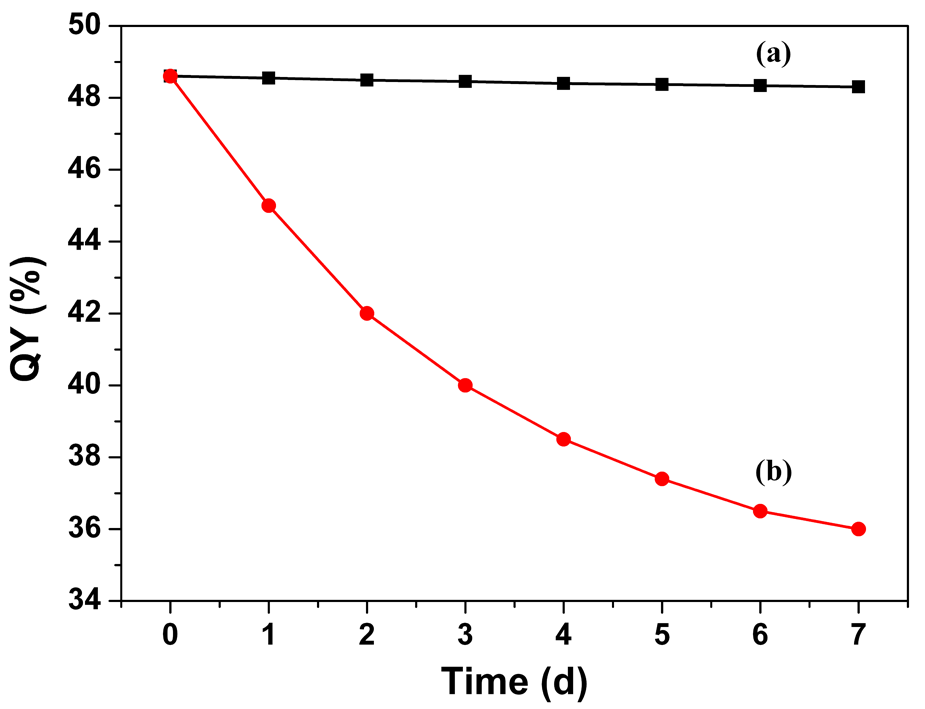
**

**Figure S3** PL quantum of (a) (FITC & RhB)/αBN, (b) FITC & RhB as function of the irradiation time, respectively.

**Fabrication process of the (FITC and RhB)/αBN-based white LEDs**

The optimized as-prepared (FITC and RhB)/αBN represented that RhB (100 mL, 1 mg/L) and FITC (100 mL, 2 mg/L) were embedded in the solid media of αBN (100 mg) at the same time. The wavelength conversion layer was prepared by mixing 6.66 g epoxy resin (CYD-128, the Baling Petrochemical Branch of Sinopec Assets Management Co.), 3.34 g amine curing agent (CYDHD-531, the Baling Petrochemical Branch of Sinopec Assets Management Co.) with (FITC and RhB)/αBN (0.2, 1, and 5 g), respectively. Accordingly, the mass ratios of (FITC and RhB)/αBN: epoxy resin were 0.2:10, 1:10, and 5:10, respectively. Finally, the composites were poured into a plastic mold with a blue LED chip (the emission wavelength of 466 nm, non-epoxy molding packages, Seoul Optodevice Co., Ltd., Korea), and cured for at least 48 h at ambient temperature for further measurements.
